# Prognostic Significance of β-Catenin Expression in Osteosarcoma: A Meta-Analysis

**DOI:** 10.3389/fonc.2020.00402

**Published:** 2020-04-09

**Authors:** Xiaoliang Xie, Yumei Li, Haixia Zhu, Zhixing Kuang, Deta Chen, Tianyou Fan

**Affiliations:** ^1^Department of Orthopedics, Shanghai Municipal Hospital of Traditional Chinese Medicine, Shanghai University of Traditional Chinese Medicine, Shanghai, China; ^2^Department of Radiotherapy, The Affiliated Nanping First Hospital of Fujian Medical University, Fujian, China

**Keywords:** β-catenin, osteosarcoma, prognosis, metastasis, overall survival

## Abstract

**Background:** β-catenin plays a crucial role in the progression of osteosarcoma. However, the clinical significance of β-catenin over-expression in osteosarcoma still remains unclear. Thus, we performed a meta-analysis of studies that evaluated the impact of β-catenin on metastasis and overall survival (OS) in osteosarcoma.

**Methods:** We searched PubMed, The Cochrane Library, Embase, Springer, Science Direct, OVID, Weipu, Wanfang and China National Knowledge Internet (CNKI) databases from their start year up to Aug.2019. Individual hazard ratios (HRs) and 95% confidence intervals (CIs) were extracted and pooled HRs with 95% CIs or odd ratio (OR) were used to evaluate the relationships between β-catenin over-expression and metastasis and overall survival in osteosarcoma.

**Results:** Eight related studies involving 521 patients were qualified for this meta-analysis. Results showed that over-expression of β-catenin was significantly correlated with metastasis (OR = 3.31, 95% CI = 2.08–5.24, *P* < 0.001) and overall survival (HR = 2.32, 95% CI = 1.48–363, *P* = 0.02).

**Conclusion:** The meta-analysis revealed that over-expression of β-catenin might be associated with distant metastasis and overall survival in osteosarcoma, which reminds that β-catenin acts as a prognostic biomarker and it can guide the clinical therapy in osteosarcoma patients.

## Introduction

Osteosarcoma is one of the most common primary malignant bone tumors in children and young adults (1), mainly arising in the metaphysis of long bones (2). It is characterized by early lung metastasis, which is the major cause of death in patients with osteosarcoma (3, 4). Although the neoadjuvant therapy with aggressive surgical resection has improved the poor prognosis (5), the treatment of osteosarcoma is still unsatisfactory mainly because of the local relapse and distant metastasis (6). Currently, means to predict osteosarcoma metastasis and prognosis can hardly be found in reported literatures, probably because carcinogenesis mechanisms remain not fully clarified and because of lack of effective indicators for prognosis (7).

Biomarkers can be used to predict the prognosis and metastasis of cancer. β-Catenin is a multifunctional protein and plays an important role in the regulation of cell proliferation, differentiation, and apoptosis. It is widely distributed in endothelial cells, fibroblasts, osteoblasts, and other types of cells (8). Plenty of studies have shown that the abnormal expression of β-catenin is associated with the occurrence and prognosis of malignant tumors such as esophageal cancer (9), lung cancer (10), breast cancer (11), and colorectal cancer (12). It indicates that β-catenin can be used as a biomarker for the prognosis of patients with malignant tumors.

Numbers of reported studies suggested that overexpression of β-catenin was associated with a high risk of distant metastasis and poor prognosis in patients with osteosarcoma (13, 14). However, other studies showed controversial results, and no consensus has been reached. To deeply understand the relationship between potential biomarkers and clinical outcomes, we conducted this meta-analysis to estimate the prognostic value of β-catenin overexpression in osteosarcoma.

## Methods

### Search Strategy and Selection Criteria

Over the internet, the PubMed, Cochrane Library, EMBASE, Springer, Science Direct, OVID, China National Knowledge Internet, Weipu, and Wanfang literature databases were searched to obtain the relevant literature published before August 2019. Articles were identified using the following search terms: “Beta-catenin, β-catenin, or CTNNB1,” “osteosarcoma, Osteogenic Sarcoma, or bone tumor,” and “prognosis, prognostic or survival.”

### Selection Criteria

The studies were included in our meta-analysis if they met the following inclusion criteria: (1) patients with osteosarcoma were diagnosed based on histopathological examination; (2) studies regarding β-catenin expression were detected using an immunohistochemistry method; (3) studies provided sufficient information on evaluation of the relationships between β-catenin expression and overall survival (OS) pathological features or prognosis; (4) sufficient information provided to estimate the hazard ratio (HR) and its 95% confidence interval (CI) and information used to calculate the HR; (5) articles were published in English or Chinese with the full text.

### Exclusion Criteria

Exclusion criteria were as follows: (1) osteosarcoma diagnosed without a biopsy and there was no clear cutoff value in the literature; (2) similar studies from the same author and multiple replicates from different literatures exclude earlier and smaller sample data; (3) animal experiments, reviews, correspondences, case reports, talks, letters, expert opinions, and editorials without original data.

### Data Extraction and Quality Assessment

Two authors evaluated the eligible studies independently according to the “Newcastle–Ottawa Scale” (NOS) (15). Each of the studies included achieved a score of more than five and was rated as qualified. Data extracted from the literature included name of first author, publication year, country, definition of β-catenin positive (cutoff), outcomes, method of HR estimation, HR and 95% CI, and method of survival analysis. If the study provided only Kaplan–Meier curves, the data were extracted using Engauge Digitizer version 4.1 software (Mark Mitchell, http://digitizer.sourceforge.net); HR and 95% CI were calculated as described by Tierney et al. (16).

### Statistical Analysis

Statistical analyses were conducted with Stata version 12.0 (stata Corp LP, www.stata.com). The HR and 95% CI were used to estimate the relationship between β-catenin expression and OS in patients with osteosarcoma, and odds ratio (OR) was used to estimate lung metastasis. The χ^2^ test and the *I*^2^ statistic were used to examine heterogeneity between selected studies. Random-effects or fixed-effects models were used based on the results of heterogeneity test; *P* < 0.1 or *I*^2^ > 50% was considered to be significant for heterogeneity, and a random-effects model was used to conduct the meta-analysis; otherwise, a fixed-effects model was used. Begg test and Egger test were used to analyze the publication bias; *P* < 0.05 was considered statistically significant. All the *P* values were used for a two-sided test with significance at *P* < 0.05.

## Results

### Study Characteristics and Quality Assessment

**Figure 1:**
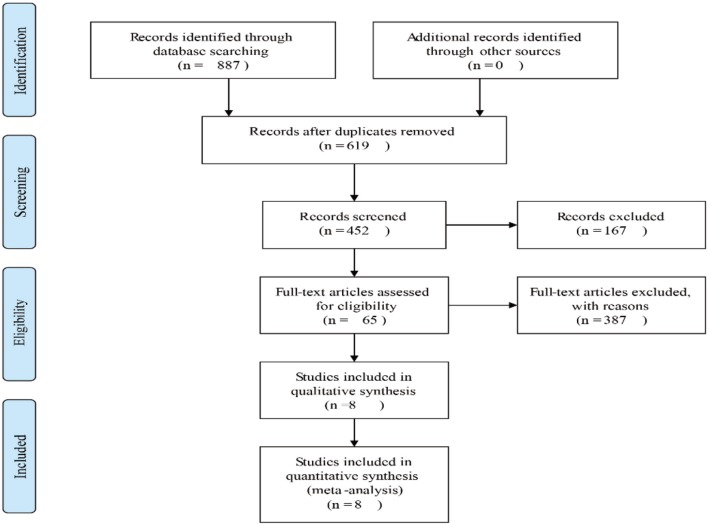
Flow diagram of the study selection in this meta-analysis.

Eight hundred eighty-seven potential relevant studies were searched from the literature databases according to the search terms. Two hundred sixty-eight duplicate reports were excluded. After scrutinizing the abstracts and full text of these studies, eight studies were ultimately met in this meta-analysis (13, 17–23) (Figure 1). The main characteristics of the included studies are summarized in Table 1. The studies in our meta-analysis were published between 2000 and 2019. A total of 521 osteosarcoma patients were included, and the relationship between β-catenin expression and pathological features or OS was investigated. All studies were of good quality with NOS scores ≥5.

**Table 1:** Characteristics of studies included in the metastasis meta-analysis.

**References**	**Region**	**Total subjects (Male/Female)**	**Testing methods**	**β-catenin Cut-off**	**β-catenin positive**		**β-catenin negative**		**HR estimation**	**HR(95%CI) OS**	**Survival**	**NOS scores**
					**Metastasis**	**Total**	**Metastasis**	**Total**				
Lu et al. (17)	China	96 (52/44)	IHC	>4 score	42	59	13	37	Sur-curve	2.06 (1.11–3.85)	OS	6
Haydon et al. (13)	USA	47 (28/19)	IHC	>0%	5	33	3	14	NA	NA	NA	7
Bao et al. (18)	China	108 (67/41)	IHC	>4 score	52	62	7	46	Sur-curve	6.36 (1.31–30.86)	OS	5
Deng et al. (19)	China	90 (53/37)	IHC	>6 score	37	54	13	36	Sur-curve	2.88 (1.35–6.13)	OS	6
Yi et al. (22)	China	36 (20/16)	IHC	>10%	11	27	2	9	Sur-curve	0.36 (0.05–2.59)	OS	7
Bi et al. (21)	China	54 (30/24)	IHC	>10%	15	37	6	17	NA	NA	NA	5
Li et al. (20)	China	35 (21/14)	IHC	>0%	15	20	3	15	NA	NA	NA	7
Liu (23)	China	55 (38/17)	IHC	>1 score	13	41	0	14	NA	NA	NA	6

*IHC, immunohistochemistry; NA, not applicable; NOS, Newcastle–Ottawa Scale; OS, overall survival*.

### Relationship Between β-Catenin Overexpression and Metastasis in Patients With Osteosarcoma

**Figure 2:**
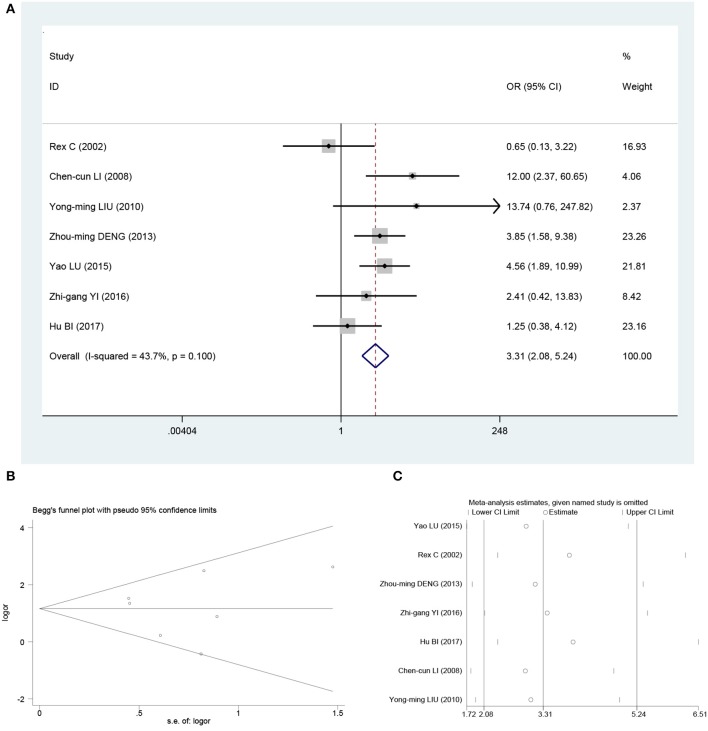
Pooled analysis for the association between β-catenin overexpression and metastasis. **(A)** Forest plots. **(B)** Funnel plots. **(C)** Sensitive analysis. OS, overall survival; OR, odds ratio; CI, confidence intervals; s.e., standard error.

Heterogeneity was significant among the included studies when assessing relationship between β-catenin overexpression and metastasis for osteosarcoma (***I***^**2**^ = 71.7%; ***P*** = 0.001). Meta-analysis with random-model β-catenin overexpression was associated with metastasis in osteosarcoma patients (OR, 4.34; 95% CI, 1.83–10.34; *P* < 0.001). To explore the heterogeneity, the literature that has a significant impact on heterogeneity is mainly in the study of Bao et al. (18). After removing this literature, the heterogeneity was reduced to *I*^2^ = 43.7% (Figure 2A); meta-analysis with fixed-effects model β-catenin overexpression was associated with metastasis in osteosarcoma patients (OR, 3.31; 95% CI, 2.08–5.24; *P* < 0.001; Figure 2A). Although the pooled effect values were decreased, the results have not changed significantly. These results suggest that β-catenin overexpression was an indicator of metastasis for osteosarcoma patients.

### Relationship Between β-Catenin Overexpression and OS for Osteosarcoma

**Figure 3:**
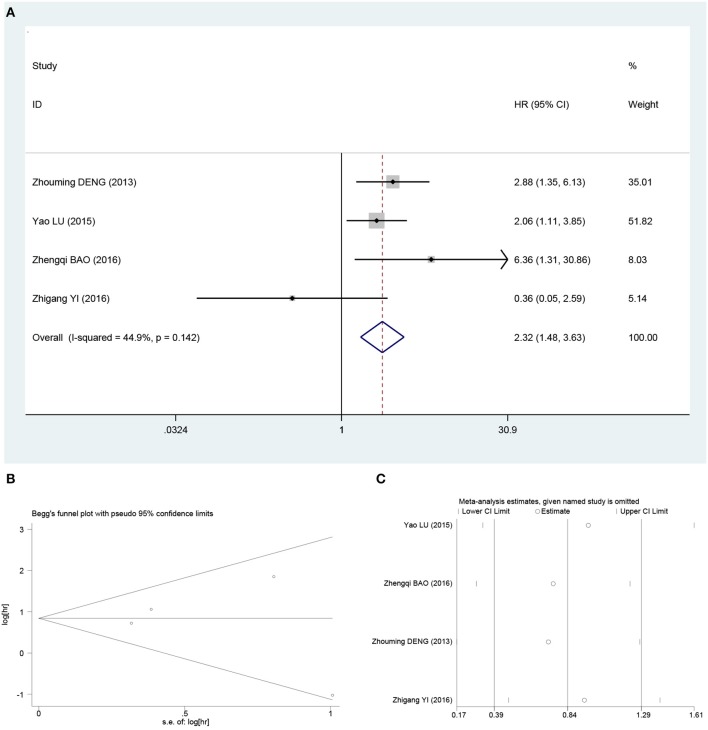
Pooled analysis for the association between β-catenin overexpression and OS. **(A)** Forest plots. **(B)** Funnel plots. **(C)** Sensitive analysis. OS, overall survival; OR, odds ratio; CI, confidence intervals; s.e., standard error.

The OS rate was extracted from four studies (17–19, 22). The fixed-effects model was used to analyze the prognostic value of β-catenin expression in osteosarcoma based on heterogeneity test results (*I*^2^ = 44.9%; *P* = 0.142). Meta-analysis indicated that β-catenin overexpression was associated with poor OS in osteosarcoma patients (HR, 2.32; 95% CI, 1.48–3.63; *P* = 0.02; Figure 3A).

### Publication Bias

Begg funnel plot and Egger test were performed to evaluate the publication bias for metastasis and OS in osteosarcoma patients included the studies. As shown in Figures 2B, 3B, there was no obvious publication bias for metastasis (Begg test of *Z* = 0.15, *P* = 0.881; and Egger test of *t* = 1.14, *P* = 0.307; Figure 2B) and OS (Begg test of *Z* = −0.52, *P* = 0.602; and Egger test of *t* = −0.31, *P* = 0.788; Figure 3B).

### Sensitivity Analysis

In order to assess the impact of individual studies on pooled HR and OS due to significant heterogeneity, we performed sensitivity analysis by estimating the average HRs in the absence of each study. The results demonstrated that our meta-analysis was statistically reliable (Figures 2C, 3C).

## Discussion

Osteosarcoma is the most common primary malignant tumors of the bone in children and young adults (1). It is propensity to lung metastasis in the early stage, with poor prognosis and poor quality of life after surgery (4) and has even become a global health problem (8). Despite the advancement of medical technology in recent years, and the 5-year survival rate of osteosarcoma patients increasing from 20% to ~65% to 75%, there are still ~70 to 80% of patients inevitably die of lung metastasis caused by chemotherapy resistance (24). Investigating and interfering with the invasion and metastasis of osteosarcoma at the molecular level can help us overcome the chemotherapy resistance and metastasis rate of osteosarcoma and are significant to improve the prognosis of osteosarcoma patients. β-Catenin is associated with unfavorable prognoses in many human malignancies (25). β-Catenin was overexpressed in tissues such as esophageal cancer (9), lung cancer (10), breast cancer (11), and colorectal cancer (12) and is associated with poor prognosis in patients (26). However, the role of β-catenin in the prognosis of patients with osteosarcoma remains controversial, and there is no unified conclusion yet. Therefore, this study attempts to elucidate the relationship between β-catenin overexpression and osteosarcoma prognosis by analyzing the results of large sample studies.

The invasion and metastasis of tumors are a complex biological process in which β-catenin plays a crucial role (12). β-Catenin is a multifunctional protein, which exists in cells in two forms: (1) binding type, in which β-catenin binds to the inner surface of cell membrane with E-cadherin to form E-cadherin/catenin complex, which mediates the adhesion between homologous cells; (2) free type, in which β-catenin participates in the Wnt signal transduction pathway, enters the nucleus through nuclear membrane, binds to LEF/TCF, and activates Wnt pathway target genes, such as c-Myc, cyclin D1, MMP-7, and CD44; all those genes play an important role in the development of tumors (27–29). Numerous of studies have also shown that high expression of β-catenin is associated with lung metastasis and poor prognosis in patients with osteosarcoma and shows poor prognosis (18, 19, 30). Furthermore, inhibition of β-catenin expression can inhibit the growth and lung metastasis of osteosarcoma xenografts in nude mice (31–33). Therefore, targeting β-catenin is a promising strategy for osteosarcoma (34).

In this meta-analysis, the association between β-catenin overexpression and OS or metastasis in patients with osteosarcoma was comprehensively reviewed. Overexpression of β-catenin was significantly associated with metastasis and contributes to distant metastasis (OR, 3.31; 95% CI, 2.08–5.24; *P* < 0.001), which is consistent with the research by Yao et al. (17). Nevertheless, it also has been reported that overexpression of β-catenin is associated with poor prognosis of osteosarcoma (13). We subsequently analyzed the relationship between β-catenin expression and OS prognosis in patients with osteosarcoma. Our result indicated that overexpression of β-catenin was a risk factor for poor prognosis in patients with osteosarcoma (HR, 2.32; 95% CI, 1.48–3.63; *P* = 0.02), favoring the hypothesis that β-catenin overexpression is associated with a poor prognosis in patients with osteosarcoma.

## Conclusion

This meta-analysis suggests that overexpression of β-catenin was associated with distant metastasis. Simultaneously, the patients with β-catenin overexpression compared with low-expression showed poorer prognosis, which suggests that β-catenin may be used as a prognostic biomarker to guide the clinical therapy in osteosarcoma. Moreover, overexpression of β-catenin can be used as one of the risk factors for assessing early lung metastasis and survival prognosis in patients with osteosarcoma. And we expect more new molecular targeted therapies in osteosarcoma.

## Limitations

This meta-analysis has some limitations. First, studies published only in English or Chinese are included in this meta-analysis, which may affect our conclusion. Second, the sample size of individual studies included in the study is small. Third, the β-catenin expression was detected by immunohistochemical staining and different detection reagents, and immunohistochemical cutoff values for β-catenin–positive expression in each article may be different, which may lead to some heterogeneity in this article. However, this conclusion still requires more and large-sample positive–negative control studies to obtain more precise and sufficient results.

## Data Availability Statement

All datasets generated for this study are included in the article/supplementary material.

## Author Contributions

XX and TF contributed to the conception and design of the research. XX and ZK contributed to acquisition of data. XX, HZ, and DC contributed to data analysis and the interpretation and completion of the figures and tables. YL and TF contributed to revision of manuscript for important intellectual content. All authors read and approved the final manuscript.

### Conflict of Interest

The authors declare that the research was conducted in the absence of any commercial or financial relationships that could be construed as a potential conflict of interest.
